# Arachidonic acid-induced Ca^2+^ entry and migration in a neuroendocrine cancer cell line

**DOI:** 10.1186/s12935-018-0529-8

**Published:** 2018-03-02

**Authors:** Priyodarshan Goswamee, Tamar Pounardjian, David R. Giovannucci

**Affiliations:** Department of Neurosciences, University of Toledo Medical Center, 3000 Arlington Avenue, Toledo, OH 43614-2598 USA

**Keywords:** Orai, Arachidonic acid, Calcium signaling, Calcium channels, Cell migration, Neuroendocrinology, Gastro-enteropancreatic neuroendocrine tumors

## Abstract

**Background:**

Store-operated Ca^2+^ entry (SOCE) has been implicated in the migration of some cancer cell lines. The canonical SOCE is defined as the Ca^2+^ entry that occurs in response to near-maximal depletion of Ca^2+^ within the endoplasmic reticulum. Alternatively, arachidonic acid (AA) has been shown to induce Ca^2+^ entry in a store-independent manner through Orai1/Orai3 hetero-multimeric channels. However, the role of this AA-induced Ca^2+^ entry pathway in cancer cell migration has not been adequately assessed.

**Methods:**

The present study investigated the involvement of AA-induced Ca^2+^ entry in migration in BON cells, a model gastro-enteropancreatic neuroendocrine tumor (GEPNET) cell line using pharmacological and gene knockdown methods in combination with live cell fluorescence imaging and standard migration assays.

**Results:**

We showed that both the store-dependent and AA-induced Ca^2+^ entry modes could be selectively activated and that exogenous administration of AA resulted in Ca^2+^ entry that was pharmacologically distinct from SOCE. Also, whereas homomeric Orai1-containing channels appeared to largely underlie SOCE, the AA-induced Ca^2+^ entry channel required the expression of Orai3 as well as Orai1. Moreover, we showed that AA treatment enhanced the migration of BON cells and that this migration could be abrogated by selective inhibition of the AA-induced Ca^2+^ entry.

**Conclusions:**

Taken together, these data revealed that an alternative Orai3-dependent Ca^2+^ entry pathway is an important signal for GEPNET cell migration.

**Electronic supplementary material:**

The online version of this article (10.1186/s12935-018-0529-8) contains supplementary material, which is available to authorized users.

## Background

In the last decade, Orai1 and stromal interacting molecule-1 (STIM1) were identified as the long sought-after molecular players that are both necessary and sufficient to recapitulate store-operated Ca^2+^ entry (SOCE) [1–8]. This pathway was shown to be physiologically and pathophysiologically important for a variety of cell types including, lymphocytes and cancer cells [9–11]. Recently, several studies have indicated a role for Orai1 and STIM1 in migration of different types of cancer and non-cancer cells [12–21]. These observations have lead to the consensus that SOCE is important for tumor cell migration [22]. Interestingly, alternative pathways that utilize many of the same molecular constituents of SOCE have been shown to contribute to other less well-characterized modes of Ca^2+^ entry [23, 24]. However, these alternative pathways have not been adequately investigated in the context of tumor cell migration. For example, the intracellular lipid second messenger, arachidonic acid (AA) and/or its downstream metabolite leukotriene-C4 (LTC4) have been shown to induce a store-independent mode of Ca^2+^ entry via a plasma-membrane channel that is comprised of both Orai1 and Orai3 [14, 25].

Although some studies indicated that AA or its metabolites such as prostaglandins and leukotrienes might stimulate migration and epithelial to mesenchymal transition (EMT) in some cancer and non-cancer cells [26–29], a role for AA-induced Ca^2+^ entry has not been examined. Therefore, we ascertained whether SOCE and/or AA-induced Ca^2+^ entry pathways contributed to cell migration in a tumor-cell line that displayed both pathways.

Previous research from our laboratory had shown that several model cell lines of gastroenteropancreatic neuroendocrine tumors (GEPNETs) expressed mRNA message for Orai homologs and exhibited SOCE [30]. In the current study, we extended these observations to assess whether exogenously administered AA could evoke Ca^2+^ entry and/or enhancement of cell migration in BON cells, a well-characterized GEPNET cell line.

Using live cell fluorimetric imaging of Ca^2+^ dynamics in combination with pharmacological treatments, we demonstrated that Ca^2+^ entry in this cell type could be induced by artificially depleting the ER stores or by exogenous application of AA. We also demonstrated that these modes of Ca^2+^ entry could be evoked and perturbed selectively. We identified the Orai channels that contributed to these two Ca^2+^ entry pathways using shRNA-mediated gene knockdown. These studies revealed that expression of Orai1 was required for SOCE and that the AA-induced pathway required both Orai1 and Orai3. Furthermore, we assessed the relative roles of SOCE and AA-induced Ca^2+^ entry in BON cell migration using a modified Boyden chamber assay. Selective stimulation or perturbation of these Ca^2+^ entry modes using a variety of pharmacological and molecular tools revealed that under our experimental conditions the AA-induced Ca^2+^ entry was the dominant Ca^2+^-signal responsible for BON cell migration. Our results suggest that Ca^2+^ entry through an Orai3-containing channel is a novel signal for BON cell migration and identifies this pathway as a potential target to limit recurring GEPNET metastasis.

## Methods

### Materials

Cyclopiazonic acid (CPA) was purchased from Calbiochem. Arachidonic acid was obtained from MP Biomedicals. Thapsigargin and SK&F 96365 (SKF) were purchased from Tocris Bioscience. Ketoprofen, ethylene glycol tetraacetic acid (EGTA) and 2-aminoethoxydiphenyl borate (2-APB) were obtained from Sigma. Leukotriene C_4_ (LTC4) and Nordihydroguaiaretic acid (NDGA) were purchased from Cayman chemical.

### Cell culture and transfection

BON cells were cultured in flasks with a 1:1 solution of DMEM and F12K supplemented with 10% fetal bovine serum (FBS) and 1% penicillin–streptomycin and maintained at 37 °C in a humidified incubator with 5% CO_2_. All cell culture reagents were obtained from Life Technologies, unless specifically indicated. For cell transfection, 5 × 10^6^ BON cells were electroporated with plasmid vectors containing shRNAs against Orai1 or Orai3 (OriGene Technologies) using the Amaxa nucleofector II device (Lonza) as per manufacturer’s instruction. All available shRNA constructs supplied by the manufacturer resulted in comparable knockdowns for each subunit, and the data from these experiments were pooled. Transfection efficiency was estimated to be approximately 90% for both constructs. Control cells were transfected with an identical plasmid that contained a scrambled message. Moreover, mock-transfected and untransfected cells were used as additional controls. All plasmids expressed either GFP or RFP and a gene for puromycin resistance. Transfected cells were maintained 2 days in culture medium containing 0.2 μg/ml puromycin dihydrochloride. In addition to western blotting (see below), knockdown of Orai1 and 3 were functionally verified using live cell Ca^2+^ imaging at different time points and the maximum effects were observed at 48 h after transfection.

### Western blotting

Lysates were prepared from BON cells at 48 h post-transfection by extraction of cellular proteins in RIPA buffer (containing 25 mM Tris–HCl, pH 7.6, 150 nM NaCl, 1% Triton X-100, 1% sodium deoxycholate, 0.1% SDS). The cell lysates were concentrated and desalted using the YM-100 Microcon centrifuge filter (Sigma Aldrich). Approximately, 50 μg of concentrated cell lysates were run on 8% Bis–Tris gels under denaturing conditions. Following separation, the protein bands were transferred onto polyvinylidene fluoride (PVDF) membranes for 90 min using the Criterion blotter (Biorad) under semi-wet conditions. The membranes were then incubated for 2 h in a solution of 5% non-fat dry milk in TBS-T buffer (containing in mM, 20 Tris/HCl, 150 NaCl and 0.1% Tween-20) to block non-specific interactions. The membranes were then cut in two and incubated overnight at 4°C with rabbit polyclonal antisera against Orai1 or Orai3 (Prosci). The membranes were washed with TBS-T buffer and probed for 2 h using an HRP-conjugated anti-rabbit secondary antibody (Millipore). Finally, the blots were washed with TBS-T buffer and treated for chemiluminescence visualization using the ECL Western Blotting substrate (Pierce). Detection of protein bands was performed using a LAS 3000 imaging system (Fujifilm). After matching the position of the bands of interests with respect to the molecular mass reference ladder (Invitrogen), the membranes were stripped using Restore solution, (Pierce) and re-probed with HRP-conjugated mouse monoclonal antibody against β-actin (Abcam) and visualized by chemiluminescence. Quantification of band density was achieved using NIH ImageJ software. The intensity of bands for Orai1 and Orai3 were comparable in untransfected, mock transfected and scrambled shRNA-transfected BON cells.

### Live cell imaging

Changes in cytosolic Ca^2+^ levels in live cells were performed using live-cell fluorescent imaging as described previously [30]. Briefly, BON cells cultured on glass coverslips were loaded with a 2 μM fura-2 AM solution prepared in a physiological saline (containing in mM 140 NaCl, 5 KCl, 2.2 CaCl_2_, 1 MgCl_2_, 10 HEPES, and 5 glucose, pH 7.4) for 30–40 min at room temperature. Changes in intracellular Ca^2+^ were represented as the ratio of fura-2 fluorescence at 510 nm evoked by sequential excitation at 340 and 380 nm at a frequency of 1 Hz. Typically, for live cell imaging experiments changes in Ca^2+^ for 20–40 cells were independently monitored and analyzed. For each experiment, 2–3 coverslips were analyzed and averaged results from at least three independent experiments were used perform statistical analysis.

### Mn^2+^ quench assay

Rate of Ca^2+^ entry independent of Ca^2+^ release or buffering was estimated by monitoring the rate of quenching of the fura-2 fluorescence signal excited at 360 nm in response to Mn^2+^ entry as previously described [30]. The rate of Mn^2+^ quench of the fura-2 fluorescence in response to pharmacological stimulation was compared with the basal rate under the unstimulated condition. The fold change in the rates of quenching from basal values was determined.

### Migration assay

BON cell migration was assessed as described previously using a modified Boyden chamber assay [31]. Briefly, 5.0 × 10^4^ BON cells were re-suspended in serum free media (SFM) and were seeded into the upper chamber, the bottom surfaces of which were coated with 50 μg/ml Type-1 rat-tail collagen (Corning Incorporated). The lower chamber was loaded with SFM containing AA with or without pharmacological inhibitors. In some experiments, the cells were pre-treated with 1 μM thapsigargin dissolved in Ca^2+^-free SFM for 15 min. In another experiment, the concentration of free Ca^2+^ was reduced to approximately 0.7 mM by adding EGTA to the media. The cells were allowed to migrate from the upper to lower chamber for 8–12 h. Following this time-period the number of migrated cells were counted and expressed as percentage of the total number seeded.

### Immunofluorescence

Immunofluorescence was used to identify various NMT markers in BON cells prior to or following arachidonic acid treatments. BON cells (5 × 10^4^ cells) were seeded onto glass coverslips or Boyden chamber inserts placed in a 6-well dish containing growth media. Following treatments, cells were fixed using a 4% paraformaldehyde solution and washed with phosphate buffered saline (PBS) (Life tech). Cells were then permeabilized using a 0.5% Triton X-100 solution (Sigma) for 5 min at and a blocking buffer containing 5% non-fat dry milk added for 1 h. Following PBS washes, samples were incubated overnight at 4 °C with the appropriate primary antibody in a humidified chamber. After PBS washes, glass coverslips were incubated with secondary antibodies conjugated to Alexa Fluor 488 or Alexa Fluor 546 for 1 h and mounted with Vectashield antifade medium (Vector Laboratories). F-actin was labeled using phallotoxin conjugated to Alexa Fluor 546 or 633. Images were obtained using a Leica SP5 Confocal microscope.

### Statistical analyses

The results from the live cell imaging and Mn^2+^-quench, were analyzed using one-way analysis of variance (ANOVA) and Dunnette’s tests were performed to assess significance among treatment groups. BON cell migration experiments using genetic and pharmacological manipulations were assessed by Tukey’s multiple comparison tests. The statistical significance of the western blot data was analyzed by 2-tailed t tests.

## Results

### Store-operated and AA induced Ca^2+^ entry are pharmacologically distinguishable in BON cells

**Fig. 1:**
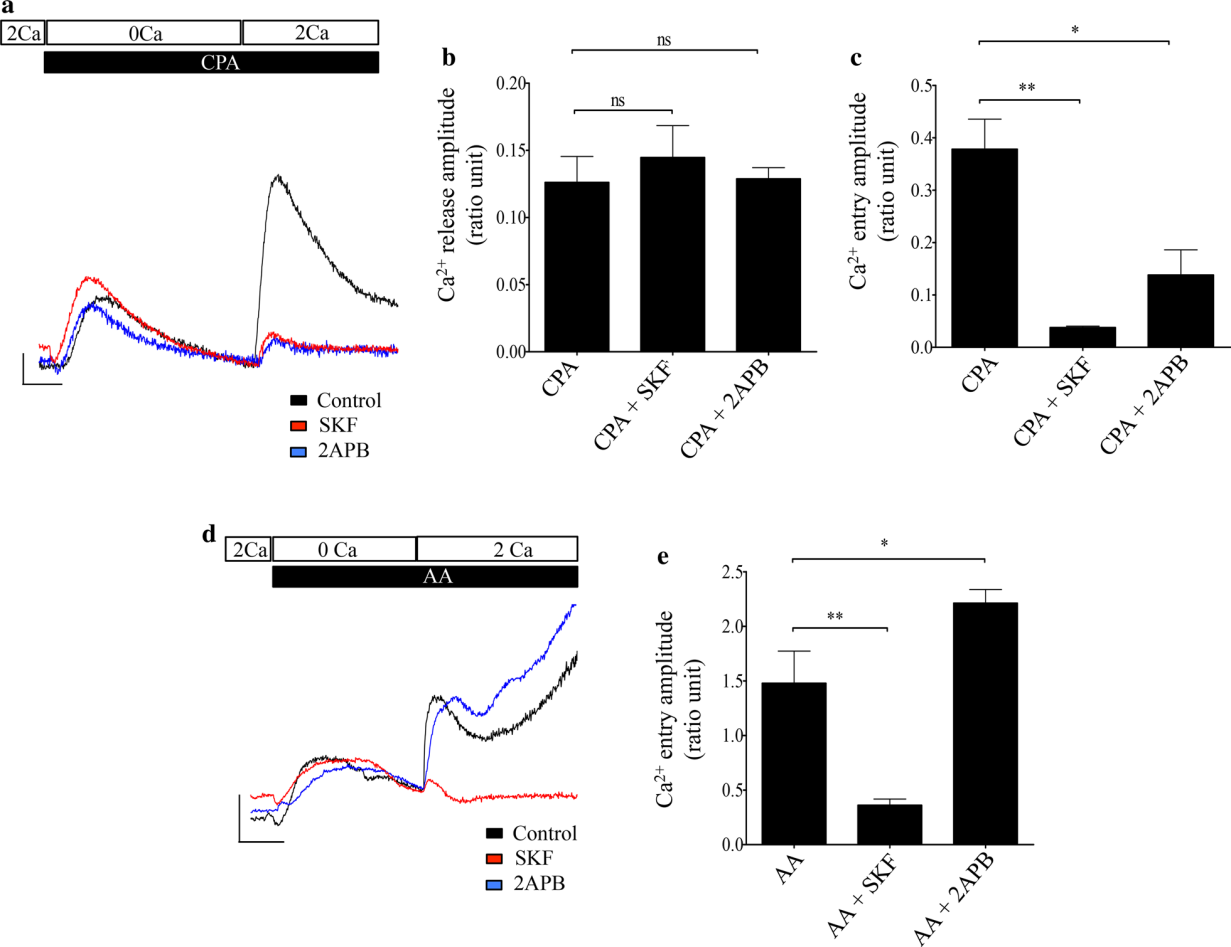
Pharmacological characterization of SOCE and AA-induced Ca^2+^ entry pathways in BON cells. **a** Representative traces showing SOCE responses in fura-2 loaded BON cells using standard protocol described in the “Methods” section. Control response is indicated by black trace. The horizontal scale bar indicates 100 s and the vertical scale bar represents a change of 0.05 ratio of fura-2 fluorescence emission (ratio units). Treatment with 30 μM SKF (red trace) or 50 μM 2-APB (blue trace) significantly attenuated this response. **b** Bar chart showing average CPA-mediated Ca^2+^ release was not altered in response to pharmacological treatments with inhibitors. **c** Bar chart showing average SOCE in response to treatments described in (**a**). **d** Representative traces showing Ca^2+^ entry evoked by application of 6 μM AA in BON cells. Control response is indicated by black trace. Treatment with 30 μM SKF (red trace) significantly inhibited the AA-induced Ca^2+^ entry. In contrast, 50 μM 2-APB (blue trace) enhanced the AA-induced response. The horizontal scale bar indicates 100 s and the vertical scale bar represents a change of 0.5 ratio units. **e** Bar chart showing averaged amplitudes of AA-induced Ca^2+^ entry in response to indicated treatments. Significance of p < 0.05 and p < 0.01 is indicated by * and **, respectively

An initial set of experiments was performed to pharmacologically characterize store-operated and arachidonic acid (AA)-activated forms of Ca^2+^ entry in BON cells by selective blockade using a broadly selective Ca^2+^ channel blocker SK&F 96365 (SKF) or 2-aminoethoxydiphenyl borate (2-APB) that has been shown to be less effective at blocking Orai3-containing Ca^2+^ channels. Store-operated Ca^2+^ entry (SOCE) was assessed using a standard protocol to deplete the endoplasmic reticulum (ER) stores of Ca^2+^ by treatment with 30 μM cyclopiazonic acid (CPA), a reversible inhibitor of the sarco-endoplasmic reticulum Ca^2+^-ATPase (SERCA). The CPA was dissolved in a saline containing nominal Ca^2+^ (0.5 mM EGTA with no added Ca^2+^). As shown in Fig. 1a, following CPA treatment there was a transient increase in concentration of cytoplasmic Ca^2+^ ([Ca^2+^]_*cyt*_) that indicated depletion of Ca^2+^ from the ER ([Ca^2+^]_*ER*_). Following restoration of 2.2 mM Ca^2+^ to the bath solution, a second, larger transient elevation in [Ca^2+^]_*cyt*_ was observed indicative of SOCE. As shown in Fig. 1a, c, activation of SOCE using standard protocol resulted in a change of 0.38 ± 0.05 ratio units (n = 3), whereas, treatment with 30 μM SKF (n = 3) or 50 μM 2-APB (n = 3) greatly attenuated the SOCE responses. The mean peak amplitudes of the response in SKF- or 2-APB-treated cells were 0.04 ± 0.00 and 0.14 ± 0.05 ratio units, respectively (n = 3). As shown in Fig. 1b, treatment with SKF or 2-APB did not have a significant effect on the store-content, as reflected by the CPA-mediated Ca^2+^ release. While, the average amplitude of the CPA-mediated release in control cells was 0.13 ± 0.02 ratio units, treatment with SKF and 2-APB resulted in Ca^2+^ release with the average amplitudes of 0.14 ± 0.02 and 0.13 ± 0.01 ratio units, respectively.

We next tested whether addition of AA activated a distinct Ca^2+^ entry pathway. Application of exogenous 1–30 µM AA induced elevations in [Ca^2+^]_*cyt*_ in a concentration-dependent manner (Additional file 1: Figure S1). A sub-maximal dose of 6 μM AA was used to treat cells for all the experiments described in the current study. As shown in Fig. 1d, application of 6 μM AA in nominal Ca^2+^-containing bath solution induced an initial transient rise in [Ca^2+^]_*cyt*_ that was followed by robust sustained elevation in [Ca^2+^]_*cyt*_ following return of Ca^2+^ in the bath solution. Typically, the secondary responses were complex waveforms and we used the peak amplitude measured within the first 300 s post-application as an index of the magnitude of the AA-induced Ca^2+^ entry. On average in about 60% of the cells, treatment with AA resulted in Ca^2+^ response with bimodal kinetics, while the other 40% of cells responded with a gradual rise and sustained increase in [Ca^2+^]_*cyt*_. These responses were in contrast to those evoked by SOCE that showed only a transient rise in cytosolic calcium. It was unclear as to what underlies the difference between these populations. Examples of these AA-induced responses are shown in Additional file 1: Figure S1. The average change in amplitude for all the experiment was 1.48 ± 0.29 ratio units (n = 3). To further distinguish AA-induced Ca^2+^ entry from SOCE we assessed the effects of known pharmacological inhibitors of SOCE. As shown in Fig. 1d, e, the AA-induced rise in [Ca^2+^]_*cyt*_ was substantially diminished by 30 μM SKF with average change in amplitude of 0.36 ± 0.06 ratio units (n = 4). In contrast, the AA-induced rise in [Ca^2+^]_*cyt*_ was not diminished by treatment with 50 μM 2-APB and was 2.21 ± 0.12 ratio units (n = 3), demonstrating that the SOCE and the AA-induced Ca^2+^ entry pathways were pharmacologically distinguishable.

**Fig. 2:**
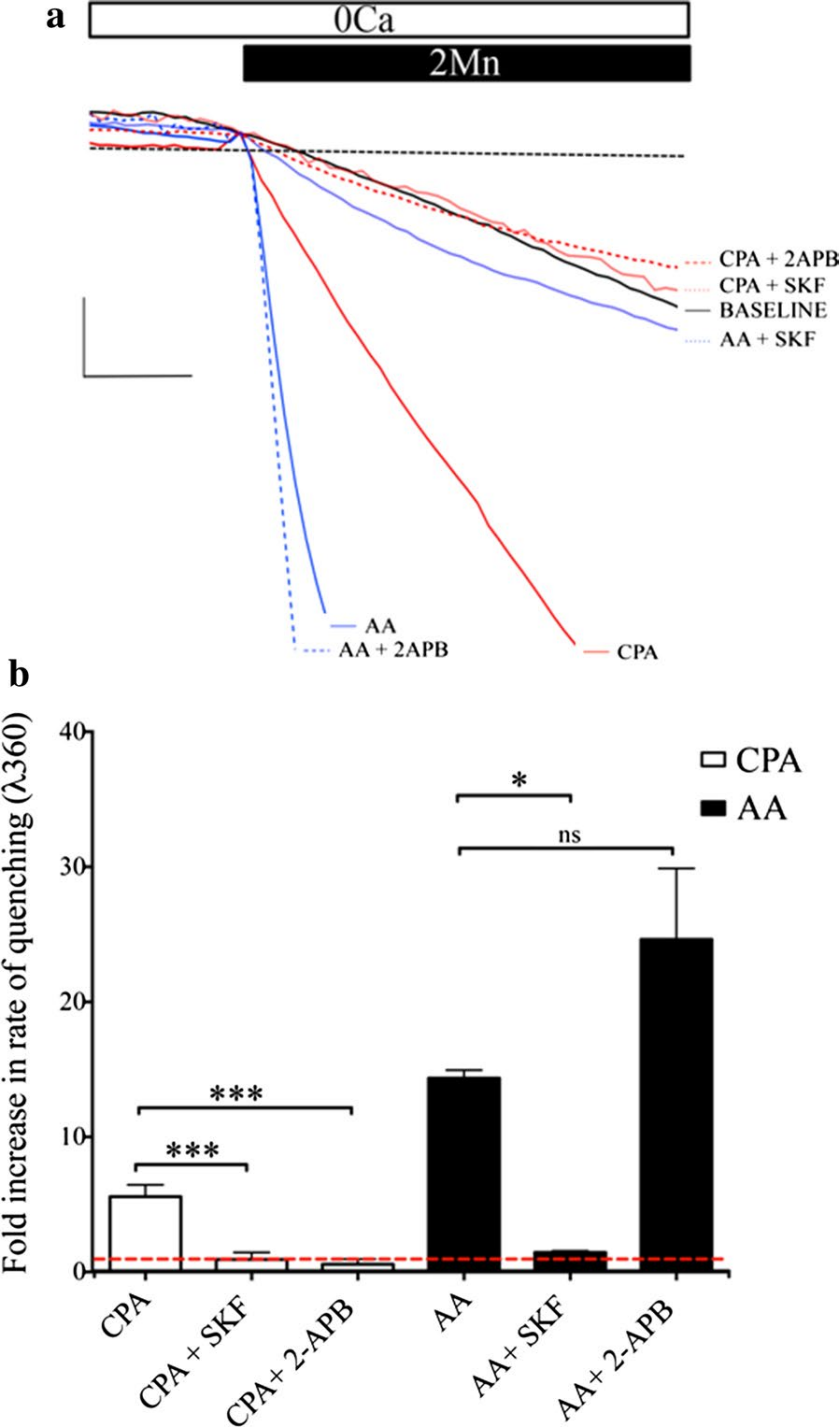
Comparison of pharmacological blockers of SOCE on AA- and CPA-activated Mn^2+^-quench. **a** Representative traces showing quenching of fura-2 fluorescence (at 360 nm excitation) by Mn^2+^ entry in response to activation of SOCE and AA-treatment with or without pharmacological inhibitors (SKF and 2-APB). **b** Bar chart showing average fold-change over baseline or unstimulated entry (red dashed line) for treatments indicated in (**a**). Significance of p < 0.05 and p < 0.001 is indicated by * and ***, respectively

We next performed a series of manganese quench assays to measure the rates of quenching and follow divalent ion entry independent of potential contribution by Ca^2+^ buffering, clearance or release from internal stores. The rates of the Mn^2+^-induced quench of the fluorescence response following activation or inhibition of SOCE and the AA-induced responses was determined and compared against the rate of quenching in unstimulated cells. As shown in Fig. 2a, b, CPA-induced store depletion resulted in a nearly sixfold faster rate of quenching than that measured in unstimulated cells. The normalized rate of quenching for unstimulated cells was 1.00 ± 0.10 fluorescent unit (FU)/s (n = 4), whereas that after CPA treatment was 5.56 ± 0.87 FU/s (n = 4). Treatment with both SKF, as well as, 2-APB diminished the rate of quenching compared to the unstimulated cells to 0.94 ± 0.50 FU/s (n = 3) and 0.58 ± 0.37 FU/s (n = 4), respectively. Treatment with 30 µM SKF or 50 µM 2-APB alone had no effect on the unstimulated rate of entry (data not shown). Consistent with the activation of a Ca^2+^ entry channel, treatment with AA resulted in rapid quenching of the fura-2 fluorescence signal. In this case the rate of quenching was 14.36 ± 0.59 FU/s (n = 4), indicating that the rate of entry was approximately 2.5 fold faster than the CPA-evoked rate of entry, and 14 times faster than the unstimulated rate of entry. In contrast to the CPA-treated cells, 2-APB treatment had no significant effect on the rate of AA-induced entry, and the rate of quenching was 24.62 ± 5.26 FU/s (n = 4). Treatment with SKF however, resulted in significant diminishment of entry. The normalized rate of AA-induced quenching was reduced to 1.45 ± 0.12 FU/s (n = 4) by treatment with SKF.

### Arachidonic acid induced Ca^2+^ release from ER but did not activate SOCE

**Fig. 3:**
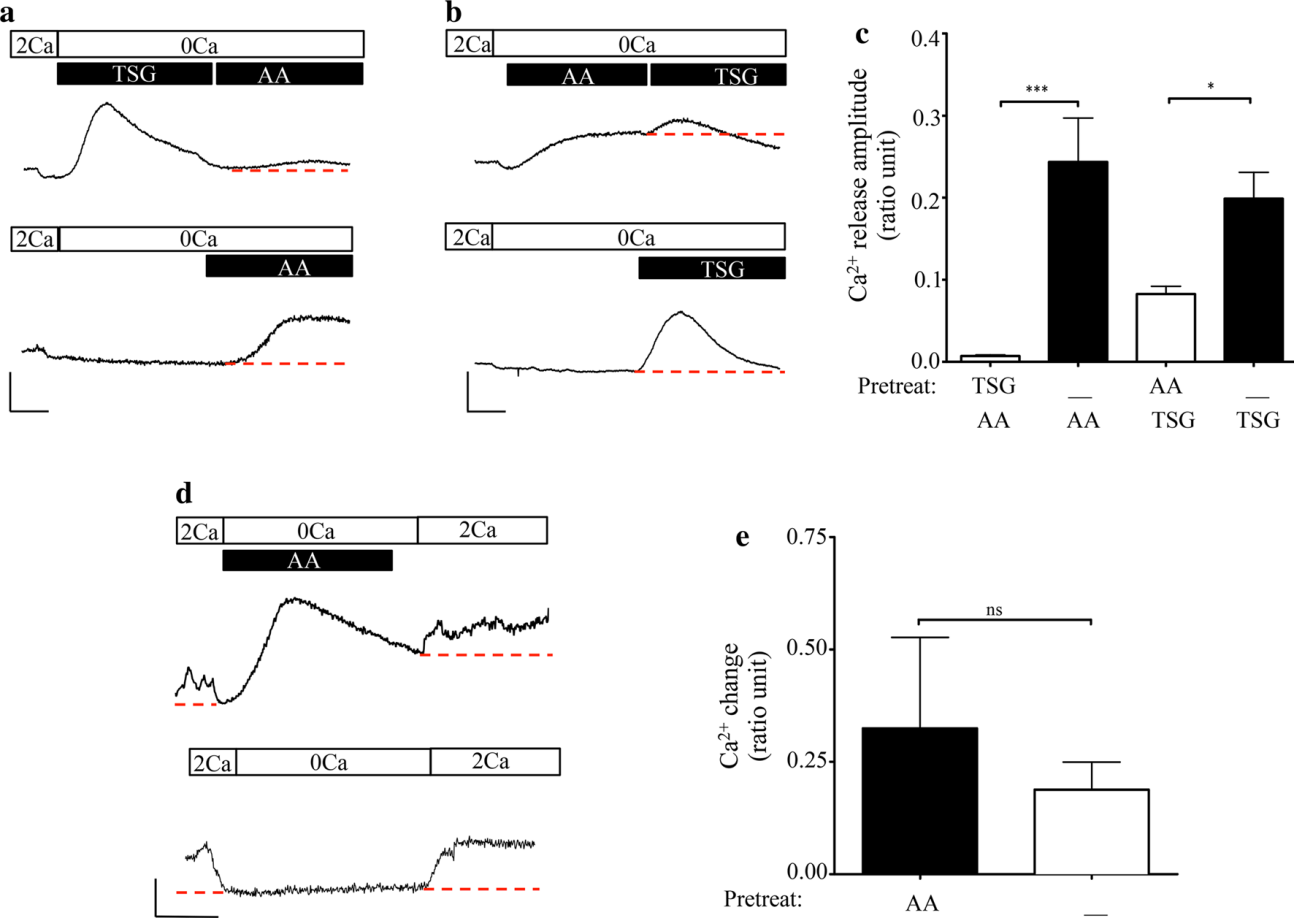
Treatment with AA depleted [Ca^2+^]_*ER*_, but was not sufficient to evoke SOCE. **a** Upper panel: representative trace showing pre-treatment of fura-loaded BON cells with 1 μM TSG in nominal Ca^2+^-containing media abolished AA-mediated release. Lower panel: representative time-matched control trace where TSG pre-treatment was omitted. Scale bars indicate 100 s (horizontal) and 0.1 ratio units (vertical). **b** Upper panel: representative trace showing pre-treatment with 6 μM AA in nominal Ca^2+^-containing media significantly diminished the TSG-mediated release. Lower panel: representative time-matched control trace where pre-treatment with AA was omitted. Scale bars indicate 100 s and 0.1 ratio units. **c** Bar chart showing average data for treatments indicated in panels **a** and **b**. **d** Upper panel: representative trace showing the effect of pre-treatment with 6 μM AA in nominal Ca^2+^-containing media on Ca^2+^ entry. Lower panel: representative time-matched control trace where pre-treatment with AA was omitted. Scale bars: x-axis = 100 s, y-axis = 0.1 ratio units. **e** Bar chart showing average change in cytosolic Ca^2+^ levels in response to change in extracellular Ca^2+^ concentration. In figures **a**, **b** and **d** magnitude of Ca^2+^ responses were measured by determining change in amplitude with respect to the red dashed line. Significance of p < 0.05 and p < 0.001 is indicated by * and ***, respectively

As shown in Fig. 1d, treatment with AA induced a rise in [Ca^2+^]_*cyt*_ in the absence of extracellular Ca^2+^. Therefore, we asked if the rise in [Ca^2+^]_*cyt*_ resulted from ER Ca^2+^ release and whether this release was sufficient to depleted the [Ca^2+^]_*ER*_ and trigger SOCE. To determine whether AA-induced Ca^2+^ release from the ER, we pre-treated the cells in nominal Ca^2+^ containing saline with thapsigargin (TSG), an irreversible inhibitor of the SERCA pump, and then applied AA. As shown in Fig. 3a (upper panel), 1 μM TSG induced robust, transient release of Ca^2+^ from ER stores. Subsequent application of AA did not induce significant additional release. The mean amplitude of this signal was only 0.007 ± 0.001 ratio units (n = 4). In contrast, in time-matched control experiments (lower panel), where TSG treatment was omitted, AA evoked Ca^2+^ release with mean amplitude of 0.24 ± 0.05 ratio units (n = 4), a value similar in magnitude to TSG-evoked release. These results indicated that AA mobilized Ca^2+^ release from a TSG- or CPA-sensitive intracellular store of Ca^2+^. Thus, to test whether AA treatment depleted [Ca^2+^]_*ER*_, a signal that is necessary to trigger canonical SOCE, we reversed the sequence of treatment as shown in Fig. 3b (upper panel). When AA was applied first, the [Ca^2+^]_*cyt*_ gradually increased to a new steady state about 200 s. Subsequent addition of TSG resulted in an additional release of Ca^2+^ with mean amplitude of 0.08 ± 0.01 ratio units (n = 3). However, this Ca^2+^ release signal was significantly smaller than that evoked by TSG alone (on average 0.19 ± 0.03 ratio units (n = 3) as shown in a time-matched control experiment (Fig. 3b: lower panel). This demonstrated that AA mobilized [Ca^2+^]_*ER*_, but did not deplete the ER in our experimental paradigm. Moreover, mobilization of [Ca^2+^]_*ER*_ by treatment with AA was not sufficient to trigger SOCE (Fig. 3d, upper panel). As shown in Fig. 3d, e, following restoration of Ca^2+^ in the bath, the mean change in amplitude for cells that were pre-treated with AA in nominal Ca^2+^-containing solution was 0.32 ± 0.20 ratio units (n = 3) (Fig. 3d, upper panel). If pre-treatment with AA was omitted, the mean change in the signal amplitude was 0.19 ± 0.06 ratio units (n = 3). These slight changes in ratio values reflected the change in steady state between Ca^2+^-containing and Ca^2+^-free media and indicated that (1) AA-induced mobilization of [Ca^2+^]_*ER*_ did not activate Ca^2+^ entry and (2) that continuous presence of AA was needed to sustain AA-induced Ca^2+^ entry.

### Contribution of Orai1 and Orai3 to store operated and AA-induced Ca^2+^ entry

**Fig. 4:**
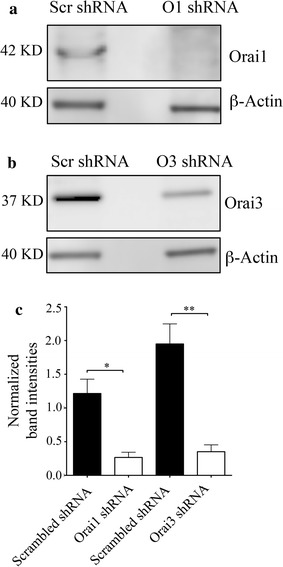
Knockdown of Orai1 and Orai3 were validated using western blotting. **a** Representative western blots showing knockdown of Orai1. **b** Representative western blots showing knockdown of Orai3. **c** Bar chart showing average Orai1 and Orai3 band intensities normalized to actin loading control for control and shRNA transfected cells. p < 0.05 and p < 0.01 are indicated by * and **, respectively

Because SOCE and the AA-induced Ca^2+^ entry pathways could be distinguished functionally in BON cells, we investigated the requirements of Orai1 and Orai3 channel subunits for these modes of Ca^2+^ entry. Specific channel subunits in BON cells were selectively knocked down by transfecting with plasmids that expressed short hairpin RNAs (shRNAs) for Orai1, Orai3 and scrambled sequences for either genes of interest as controls. Since, previous work indicated that BON cells express low levels of Orai2 mRNA, the role of this homolog in mediating Ca^2+^ entry was not assessed in the current study [30]. Western blotting was performed 48 h post-transfection to validate the effectiveness of knockdown of protein expression. As shown in Fig. 4, Orai protein band densities were normalized to the β-actin loading control and expressed as a ratio. The Orai1 expression level in cells that were transfected with scrambled shRNA was determined to be 1.21 ± 0.21 ratio units (n = 4). In contrast, when cells were transfected with Orai1 shRNA the ratio value was 0.26 ± 0.07 ratio units (n = 4) and represented approximately 78% knockdown of Orai1. Expression of Orai3 was knocked down by 82% when compared to control cells that were transfected with scrambled shRNA. (The average ratio values of the Orai3 bands in cells transfected with scrambled and Orai3 shRNAs were 1.95 ± 0.29 (n = 4) and 0.35 ± 0.10 ratio units (n = 4), respectively). In addition, cell lysates from knocked down cells were probed for the expression of both Orai1 and Orai3 homologs to test for changes in the expression of the non-targeted homolog. In these experiments, the average Orai1 ratio value of expression in cells transfected with Orai3 shRNA was 1.07 ratio units (n = 2) and the ratio value of Orai3 expression in cells transfected with Orai1 shRNAs was 1.82 ratio units (n = 2). These values were comparable to control values and indicated there was no significant effect on the expression of the non-targeted homolog. An example of this control is shown in Additional file 2: Figure S2.

**Fig. 5:**
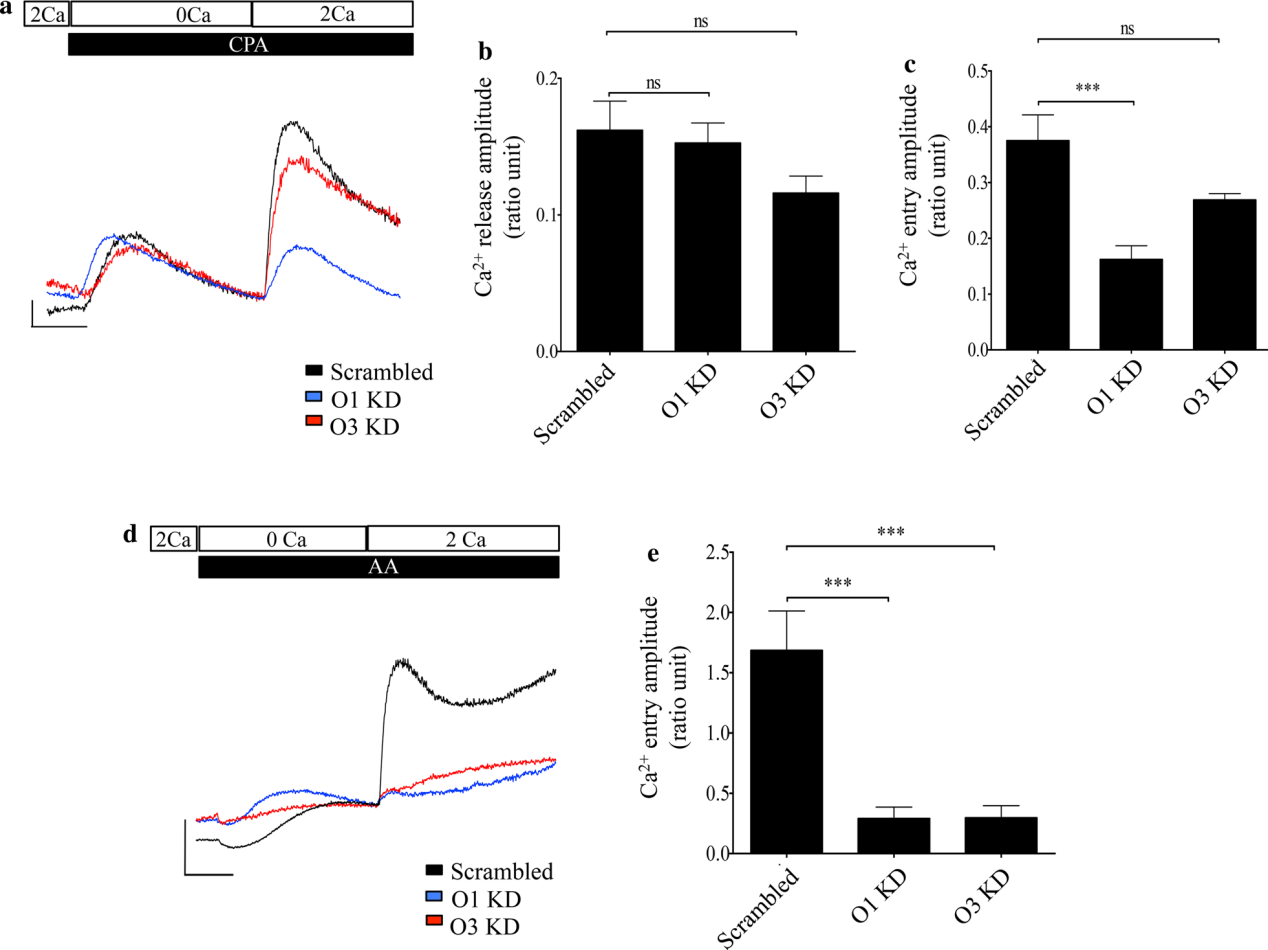
Effect of knockdown of Orai1 and Orai3 on SOCE and AA-induced Ca^2+^ entry. **a** Representative traces showing SOCE response in BON cells transfected with scrambled shRNA (black trace), Orai1 shRNA (blue trace) and Orai3 shRNA (red trace). The horizontal scale bar indicates 100 s and the vertical scale bar represents a change of 0.05 ratio units. **b** Bar chart showing average Ca^2+^ release from cells transfected with scrambled, Orai1 and Orai3 shRNAs. **c** Bar chart showing average SOCE responses in BON cells transfected with scrambled shRNA, Orai1 shRNA and Orai3 shRNA. **d** Representative traces showing AA-induced Ca^2+^ entry in BON cells transfected with scrambled shRNA (black trace), Orai1 shRNA (blue trace) and Orai3 shRNA (red trace). The horizontal scale bar indicates 100 s and the vertical scale bar represents a change of 0.5 ratio units. **e** Averaged amplitude of AA-induced Ca^2+^ entry in response to treatments indicated in (**d**). Significance of p < 0.001 is indicated by ***

The functional consequences of knockdowns of Orai homologs on Ca^2+^ entry were investigated using live cell Ca^2+^ imaging methods. As shown in Fig. 5a, c, knockdown of Orai1 significantly attenuated the amplitude of SOCE in comparison with cells transfected with scrambled shRNA. While the mean SOCE amplitude for cells where Orai1 was knocked down was 0.16 ± 0.02 ratio units (n = 5), the amplitude for cells transfected with scrambled shRNA was 0.38 ± 0.04 ratio units (n = 5). However, knockdown of Orai3 did not significantly diminish the mean SOCE amplitude. In these cells, the peak amplitude of SOCE was on average 0.27 ± 0.01 ratio units (n = 5). As shown in Fig. 5b, the genetic manipulation had no significant effect on the CPA-evoked Ca^2+^ release signal. The average amplitude of the CPA-evoked release signal in cells that were transfected with scrambled shRNA was 0.16 ± 0.02 ratio units and that for cells transfected with Orai1 and Orai3 shRNAs were 0.15 ± 0.01 and 0.12 ± 0.01 ratio units, respectively.

In contrast to the SOCE responses, knockdown of either Orai1 or Orai3 significantly decreased the amplitude of the AA-induced responses compared to cells transfected with scrambled shRNA. While the mean amplitude of the AA-induced Ca^2+^ entry in cells transfected with scrambled shRNA was 1.68 ± 0.32 ratio units (n = 5), cells that were transfected with Orai1 or Orai3 shRNAs had significantly reduced peak amplitudes of 0.29 ± 0.09 (n = 6) and 0.29 ± 0.10 (n = 6) ratio units, respectively. These results indicated that both Orai1 as well as Orai3 were required for mediating Ca^2+^ entry in response to AA and that channels that contained predominantly Orai1 were required for SOCE.

### Effects of AA-induced and store-operated Ca^2+^ entry on cell migration

**Fig. 6:**
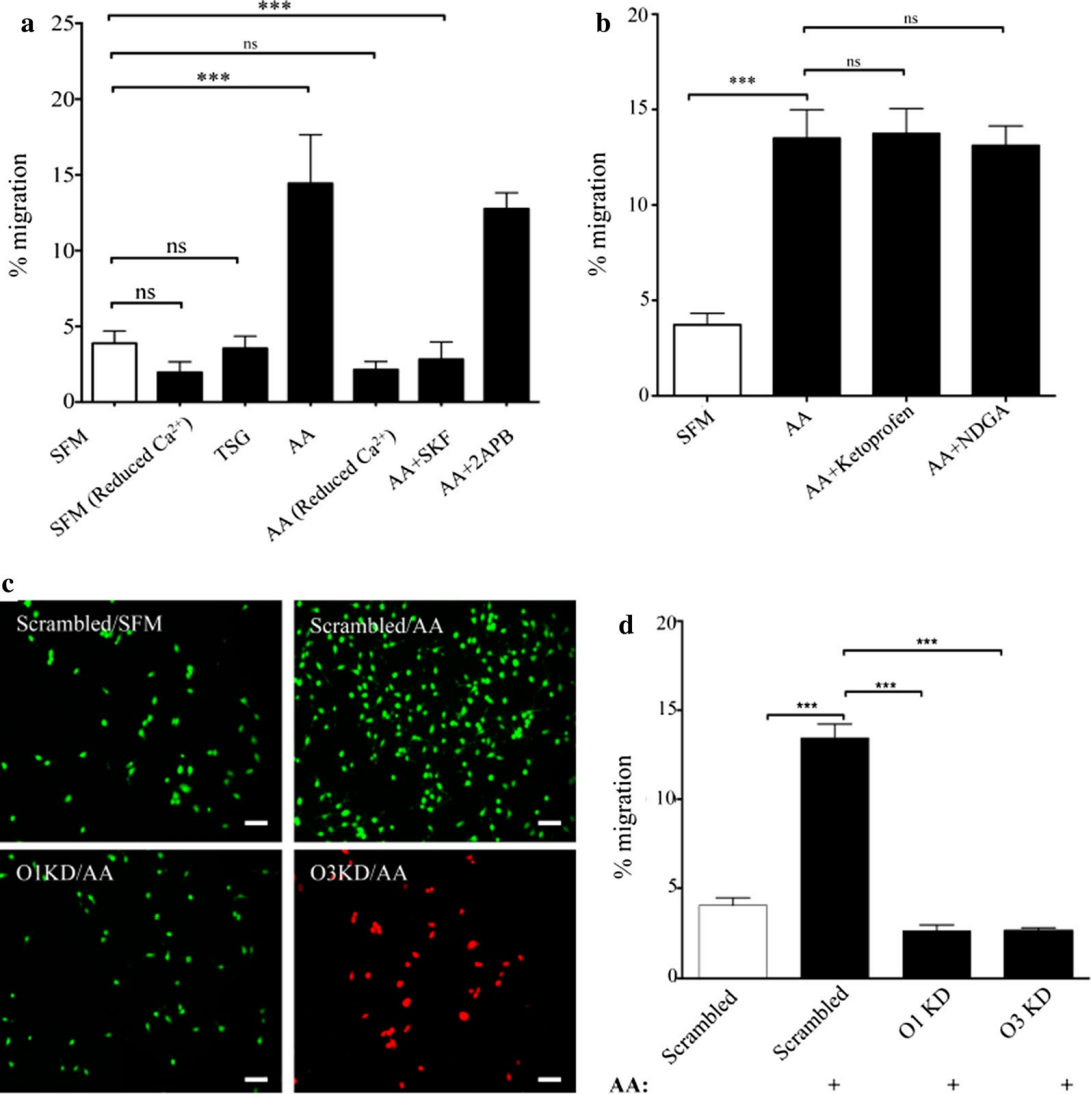
Effect of SOCE and AA-induced Ca^2+^ entry on cell migration. **a** Bar chart showing average percentage of cells that migrated in response to activation or pharmacological inhibition of SOCE and AA-induced Ca^2+^ entry (treatments indicated). **b** Bar chart showing average percentage of cell migration in response to pharmacological inhibition of AA metabolic pathways. Significance of p < 0.001 is indicated by ***. Effect of knock down of Orai1 and Orai3 on cell migration. **c** Representative fluorescent image showing migrated cells: Top left: migration of cells transfected with scrambled shRNA in response to SFM. Top right: migration of scrambled shRNA transfected cells in response to 6 μM AA. Bottom left: migration of Orai1 shRNA transfected cells in response to 6 μM AA. Bottom right: migration of cells transfected with Orai3 shRNA in response to 6 μM AA. Scale bars indicate 50 μm. **d** Bar chart showing average migration of cells in response to treatments indicated in (**c**). Significance of p < 0.001 is indicated by ***

We next selectively activated store-operated or AA-induced Ca^2+^ entry to assess their relative effectiveness to induce migration in BON cells. We first tested whether the induced cell migration required extracellular Ca^2+^. As shown in Fig. 6a, reduction of extracellular Ca^2+^ had minimal effect on the basal migration compared to SFM alone. For example, the percentage of cells migrated in SFM was 3.89 ± 0.40% (n = 4) while the percentage of cells migrated in a reduced Ca^2+^-containing SFM was 1.96 ± 0.40% (n = 3). Moreover, direct activation of SOCE by treatment of BON cells with either 1 μM TSG or 30 µM CPA failed to enhance cell migration significantly above basal levels with only 3.54 ± 0.47% cells migrated (n = 3). In contrast, migration of BON cells was enhanced approximately threefold in response to treatment with 6 μM AA where 14.43 ± 1.61% of cells migrated (n = 4). Reducing extracellular Ca^2+^ abrogated the AA-induced enhancement of cell migration (2.15 ± 0.31%; n = 3) consistent with a requirement for Ca^2+^ entry. To further assess the relative contributions of store-operated- and AA-induced Ca^2+^ entry in BON cell migration, the pharmacological inhibitors of SOCE, SKF and 2-APB, were tested on AA-induced BON cell migration. Treatment with SKF significantly reduced the AA-induced cell migration. In these experiments only 2.82 ± 0.58% cells on average migrated (n = 4). In contrast, 2-APB had no significant effect on the AA-induced enhancement of cell migration when compared to AA alone. In these experiments 12.76 ± 0.53% cells on average migrated (n = 4). It was interesting to note that in contrast to the acute effects of AA-treatment where Ca^2+^ entry was enhanced in the presence of 2-APB, inclusion of 2-APB did not enhance AA-induced cell migration. This most likely resulted from a difference in the time courses of treatment for Ca^2+^ imaging and Boyden chamber experiments. Taken together, activation of AA-induced Ca^2+^ entry, but not SOCE, could mobilize a sub-population of BON cells to migrate in a Boyden chamber.

However, because AA can be readily metabolized into other bioactive classes of compounds such as leukotrienes and prostaglandins, we assessed whether AA-metabolites contributed in BON cell migration. Moreover reports by Mohamed Trebak’s group demonstrated that leukotriene-C4 could mediate a store-independent Ca^2+^ entry via Orai1/3 channels [14, 24]. To test whether AA-metabolites were involved in BON cell migration, we treated the cells with pharmacological inhibitors of AA-metabolism. Ketoprofen, a non-selective inhibitor of cyclooxygenase I and II, and nordihydroguaiaretic acid (NDGA), a pan-lipoxygenase inhibitor were included along with AA in the migration assays. As shown in Fig. 6b, neither of these compounds caused a significant reduction in AA-induced cell migration. In presence of ketoprofen or NDGA, 13.7 ± 0.76% (n = 3) or 13.12 ± 0.59% (n = 3) cells migrated, respectively. These data were consistent with the idea that AA itself, and not its metabolite caused the enhancement of cell migration.

**Fig. 7:**
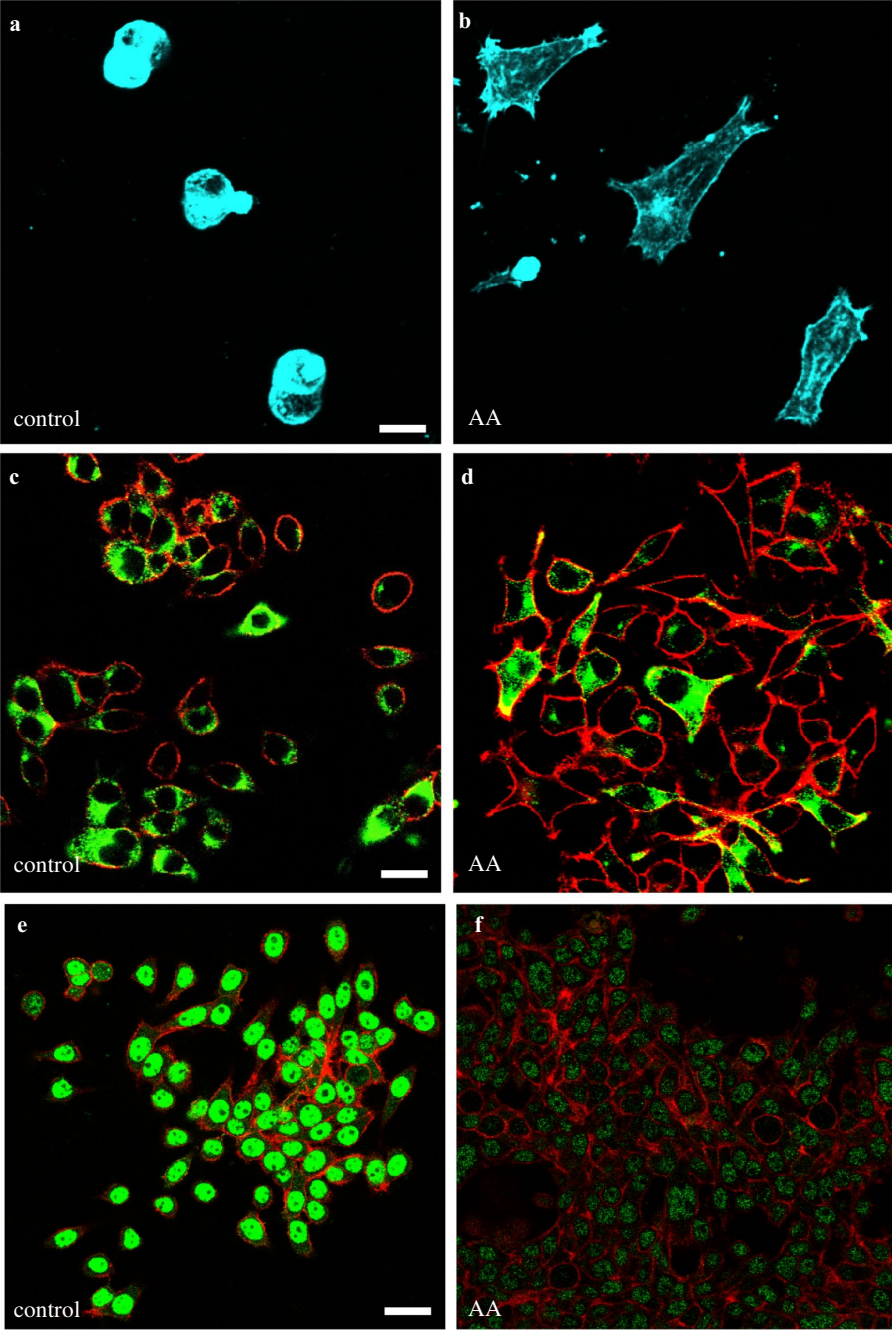
Morphological and phenotypic changes prior to or following AA treatment. **a**, **b** Confocal images of unmigrated and migrated BON cells acquired from transwell inserts following treatment with 6 µM AA for 8 h. Cell morphology was visualized by F-actin labeling with Alexa Fluor 633 conjugated phallotoxin. **c**, **d** Images of BON cell cultures following overnight treatment with vehicle alone (**c**) or with (**d**) AA treatment. Cells were labeled with antisera for CgA (green) and AlexaFluor 546 conjugated phallotoxin for F-actin (red). Percentages of cells expressing CgA were quantified under the two treatment conditions. **e**, **f** Assessment of E-cadherin expression levels (green) and F-actin (red) following overnight treatment with vehicle or AA, respectively. Each image set was obtained with the same intensity and settings to assess relative changes in fluorescence intensity evoked by treatment. Scale bars on image sets were 15, 20 and 25 µm, respectively

Because AA-induced Ca^2+^ entry is thought to be mediated by a channel that contains both Orai1 and Orai3, we investigated the role of these homologs in AA-induced cell migration. As shown in Fig. 7c, d, knockdown of either Orai1 or Orai3 in BON cells resulted in a dramatic reduction in AA-induced migration compared to cells transfected with scrambled shRNA. While 13.45 ± 0.77% (n = 3) of the cells transfected with scrambled shRNA migrated when, only 2.69 ± 0.32% (n = 3) of cells transfected with Orai1 shRNA and 2.68 ± 0.15% (n = 3) of cells transfected with Orai3 shRNA migrated in response to application of 6 μM AA. Since knocking down Orai3 had a minimal effect on SOCE, the result of this experiment was consistent with our pharmacological data that suggested that AA-induced Ca^2+^ entry is the dominant Ca^2+^ entry pathway regulating BON cell migration in vitro.

### Morphological and phenotypic changes following arachidonic acid treatment

Transformed cells can exhibit a migratory phenotype that correlates with increased metastatic potential. In BON cells that express both epithelial and neuroendocrine features, this can be achieved via neuroendocrine-to-mesenchymal transition (NMT). This transition is characterized by a reduction in neuroendocrine markers and altered expression of mesenchymal proteins [32–34]. Thus, we assessed whether AA treatment could induce morphological changes and alter expression of markers of NMT including chromogranin A (CGA) E-cadherin, α-smooth muscle actin (α-SMA) and snail family transcriptional repressor 1 (Snail1).

In these experiments, cells were plated onto coverslips or applied to transwell inserts, serum starved for 4 h, incubated overnight or for up to 48 h in growth medium supplemented with 6 µM arachidonic acid or 0.1% DMSO as vehicle control and subsequently assessed by immunofluorescence. Morphological changes induced by AA treatment were visualized by F-actin staining using fluorescently labeled phalloidin. The majority of control cells appeared clustered and were rounded or oblong in shape (~ 97%), whereas the majority of AA treated cells (~ 93%) exhibited an elongated, flattened shape that was often decorated with multiple cellular processes. Additional images of these morphologies are shown in Additional file 3: Figure S3. As indicated above, migration assays revealed that AA treatment induced migration in a subset of BON cells. Therefore, we removed and imaged chamber inserts following AA treatment and assessed the morphology of non-migrated and migrated cells using confocal microscopy. As shown in Fig. 7a, b, staining for F-actin revealed distinctive morphologies between these two populations. The unmigrated cells on the surface of the transwell insert appeared to be in clusters and exhibited rounded shapes whereas the migrated cells on the bottom of the insert typically exhibited flattened irregular shapes with processes. These respective morphologies were similar to those observed for cells cultured on glass coverslips that were treated with vehicle or AA.

One hallmark of NMT is a reduction in the neuroendocrine secretory granule protein CgA. Under our experimental conditions, approximately 34% of cells in control groups exhibited perinuclear and subplasmamembrane distribution of CgA signal. Following overnight AA treatment, less than 15% of cells showed labeling. Figure 7c, d indicated that following AA treatment there was about a 50% reduction in the number of cells expressing CgA. Moreover, the treated cells appeared much more mesenchymal in morphology and some cells showed persistent expression and ubiquitous distribution of the CgA label through out the cell including the filipodial extensions. Longer treatments (24–48 h) showed a further reduction in the number of CgA expressing cells.

Furthermore, as shown in Fig. 7e, f, there was a clear reduction in the intensity of E-cadherin staining following AA treatment. In comparing images of control and AA-treated cells, there was typically greater than 60% reduction in the mean pixel intensity of the E-cadherin (green) signal. The findings were consistent with NMT phenotype. Of note, the E-cadherin signal in BON cells had an atypical nuclear distribution, similar to that previously shown to correlate with invasiveness in pancreatic NETs [34].

In contrast, we did not observe changes in expression levels of the mesenchymal proteins α-SMA and Snail1 following AA treatment (data not shown). The lack of an effect may be in part be explained the surprising expression of these markers in BON cells prior to AA treatment.

## Discussion

Previous work from our laboratory provided molecular and functional characterization of the SOCE pathway [30] in a variety of GEPNET cell lines. It was revealed that BON cells robustly expressed messages for Orai1 and Orai3, but not Orai2. In the current study, we extended these observations to demonstrate protein expression and functional contributions of Orai1 and Orai3 channel subunits to Ca^2+^ signals and cell migration. These findings support the hypothesis that AA-induced Ca^2+^ entry through an Orai1/Orai3 containing channel was important for migration of a sub-population of BON cells.

Recent work from several groups has demonstrated a requirement of Orai1-containing channels for SOCE and cell migration in some tumor cell lines. Although knockdown of Orai1 suppressed BON cell migration, it was unlikely that the suppression was mediated by inhibition of a canonical store-operated channel. This idea was supported by the observation that direct activation of SOCE by TSG-induced store depletion was ineffective at stimulating migration. Although this finding is tempered by the caveat that prolonged treatment with TSG can induce ER stress and apoptosis.

Our contention is that it was more likely that the knockdown of Orai1 inhibited an AA-induced Ca^2+^ entry channel that contained Orai3 as well as Orai1 subunits. Consistent with this proposal, we found that treatment with AA resulted in Ca^2+^ influx that was distinct from canonical SOCE. Moreover, activation of this pathway stimulated migration of BON cells, and that when inhibited, abrogated the enhancement in migration. These data showed that Orai1 was necessary for mediating SOCE, whereas AA-induced Ca^2+^ entry as well as BON cell migration required both Orai1 and Orai3.

The AA-induced Ca^2+^ entry channel characterized here is reminiscent of the store-independent ARC channel described by Shuttleworth’s group [35, 36]. More recently, Trebak’s group identified an Orai1/Orai3-containing leukotriene-C4 (LTC4) inducible channel that mediated Ca^2+^ entry in vascular smooth muscle cells in response to thrombin stimulation [14]. Although we did not directly test whether LTC4 could activate Ca^2+^ entry or cell migration in our system, pharmacological inhibition of AA-metabolism did not suppress AA-induced cell migration. This indicated that the enhancement of cell migration observed was largely due to AA itself and not its metabolite.

Assessment of migration using pharmacological tools was also consistent with the involvement of an Orai3-containing channel. Typically, 2-APB has been used as a tool to block SOCE and has been shown in some studies to inhibit cell migration. For example, 2-APB was shown to block EGF-induced cell migration in nasopharyngeal carcinoma [21] and wound healing of clear cell renal cell carcinoma [20]. However, we found that treatment with 2-APB suppressed SOCE but not AA-induced Ca^2+^ entry or AA-evoked BON cell migration, consistent with previous work that showed 2-APB does not block, and may even enhance, conductance in Orai3-containing channels. In contrast, the broader spectrum channel blocker SKF effectively blocked Ca^2+^ entry through Orai1- and Orai3-containing channels. These findings indicated a novel mechanism by which Orai-mediated Ca^2+^ entry may contribute to migration in this biologically idiosyncratic group of cancers.

In this context, it should be noted that a mutual antagonism between SOCE- and AA-evoked Ca^2+^ entry has been observed in other cell types [37–39] and thus modes of entry may interact to set the migration potential of NET cells. Indeed, the migration potential may in part be mediated by NMT, consistent with our findings that AA treatment altered the morphology and phenotype of BON cells such that the migrated cells appeared more mesenchymal in state.

Based on our findings it is tempting to speculate that the AA-induced Ca^2+^ channel has relevance for GEPNET pathophysiology. For example, these cancers are thought to arise from neurosecretory cells in the gastrointestinal tract and commonly metastasize to the liver. Both the small intestine and liver exhibit elevated concentrations of AA [40, 41] compared to other regions of the gut. Moreover, neuroendocrine tumor cells are known to secrete several biogenic amines and peptides. Many of these secretory products have been linked with increased production of AA in different cell types. For example, serotonin has been shown to stimulate the PLA_2_-driven synthesis of AA in hippocampal neurons [42]. Bradykinin, an intestinal peptide that is secreted by neuroendocrine cells has been shown to stimulate AA synthesis in gut [43]. Moreover, we recently reported that conditioned media (CM) derived from cultured BON cells had a pro-migratory effect on these tumor cells, presumably through an autocrine mechanism [31]. Although we did not directly, test whether treatment with CM stimulated synthesis of AA in our system, unpublished experiments related to this study showed that the pharmacological inhibition of AA synthesis resulted in a decrease in CM-evoked cell migration that could be recovered by application of AA.

## Conclusions

The current manuscript demonstrates that treatment with AA induced Ca^2+^ entry through an Orai1/Orai3 heteromeric ion channel and that this mode of Ca^2+^ entry is important for migration in a GEPNET cell-line. The findings suggest that AA-induced Ca^2+^ entry may help set the migratory potential of these tumor cells and identify Ca^2+^ entry through the Orai3-containing channel as a novel signal for BON cell migration that may be exploited for therapeutic prevention of recurring GEPNET metastasis.

## Additional files

**Additional file 1: Figure S1.** Calcium entry amplitude and dynamics evoked by AA treatments. (A) Bar graph showing the peak amplitude increases in cytosolic Ca^2+^ in fura-2 loaded BON cells induced by application of different concentrations of arachidonic acid. Peak ratio changes for 1, 3, 6, 10 and 30 µM AA applications was 0.45 ± 0.19, 0.69 ± 0.24, 1.48 ± 0.18, 2.10 ± 0.27 and 4.71 ± 0.72, respectively (n = 3). B. Examples of typical Ca^2+^ dynamics induced by AA treatment. On restoration of extracellular Ca^2+^ we observed two typical responses: a more common, complex waveform (black) and a slower, gradual and sustained rise (red). Scale bars = 100 s and 0.500 ratio units.

**Additional file 2: Figure S2.** Selective knockdown of Orai channel subunit proteins. Western blot data demonstrating that silencing of one Orai channel paralog does not induce changes in expression of the other paralog. shRNAs are indicated as scrambled (Scr) or selective for Orai 1 (O1) or Orai 3 (O3). Actin expression was used as a loading control.

**Additional file 3: Figure S3.** Morphological changes induced in BON cells by overnight treatment with 6 µM AA. Cell shapes were visualized by treatment with phallotoxin fluorescently-labeled with Alexa Fluor 546 (red) or 633 (cyan). Confocal images of cells in culture are shown following treatment with vehicle contro (DMSO) or AA as indicated.
