# Supplementary figures and images for: Arachidonic acid-induced Ca2+ entry and migration in a neuroendocrine cancer cell line
Source: Cancer Cell Int. 2018 Mar 2;18:30. doi: 10.1186/s12935-018-0529-8 (PMC5834873; doi:10.1186/s12935-018-0529-8)

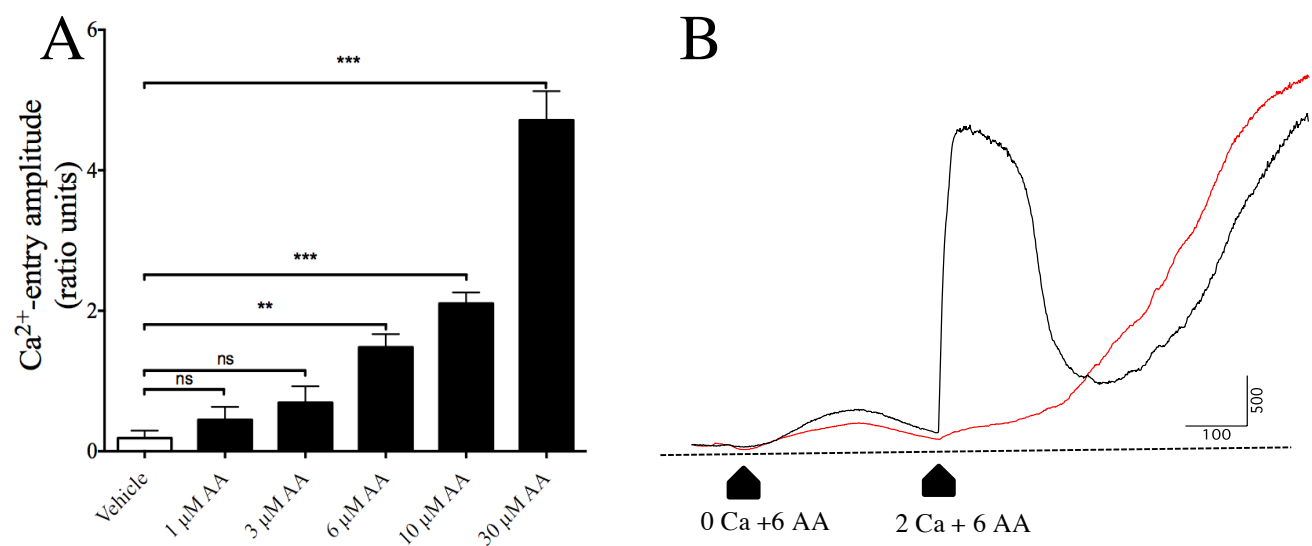

Supplementary Figure 1

Supplement: Supplementary file 1 — Additional file 1: Figure S1. Calcium entry amplitude and dynamics evoked by AA treatments. (A) Bar graph showing the peak amplitude increases in cytosolic Ca2+ in fura-2 loaded BON cells induced by application of different concentrations of arachidonic acid. Peak ratio changes for 1, 3, 6, 10 and 30 µM AA applications was 0.45 ± 0.19, 0.69 ± 0.24, 1.48 ± 0.18, 2.10 ± 0.27 and 4.71 ± 0.72, respectively (n = 3). B. Examples of typical Ca2+ dynamics induced by AA treatment. On restoration of extracellular Ca2+ we observed two typical responses: a more common, complex waveform (black) and a slower, gradual and sustained rise (red). Scale bars = 100 s and 0.500 ratio units. [file 12935_2018_529_MOESM1_ESM.pdf]

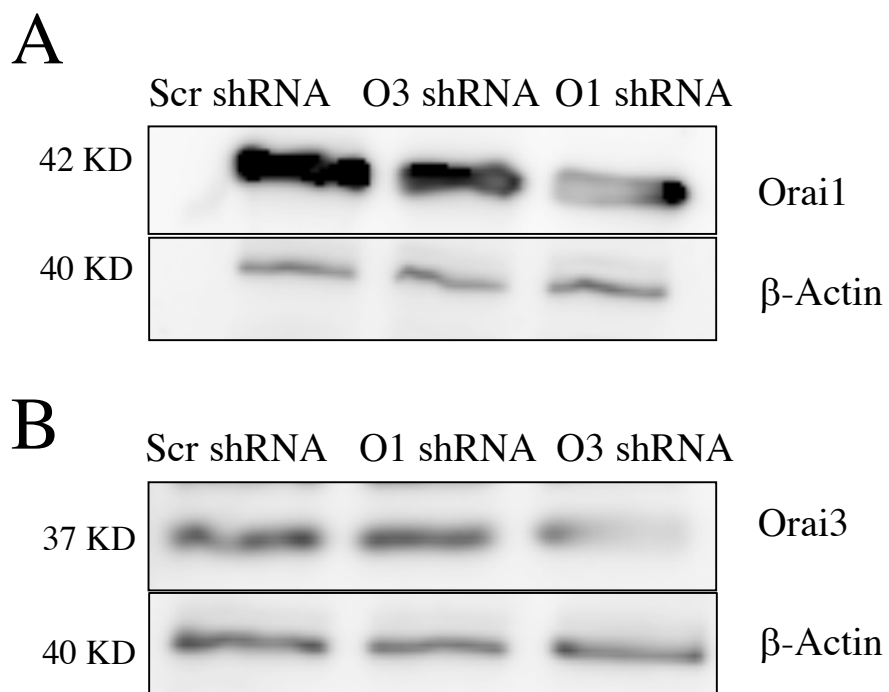

Supplementary Figure 2

Supplement: Supplementary file 2 — Additional file 2: Figure S2. Selective knockdown of Orai channel subunit proteins. Western blot data demonstrating that silencing of one Orai channel paralog does not induce changes in expression of the other paralog. shRNAs are indicated as scrambled (Scr) or selective for Orai 1 (O1) or Orai 3 (O3). Actin expression was used as a loading control. [file 12935_2018_529_MOESM2_ESM.pdf]

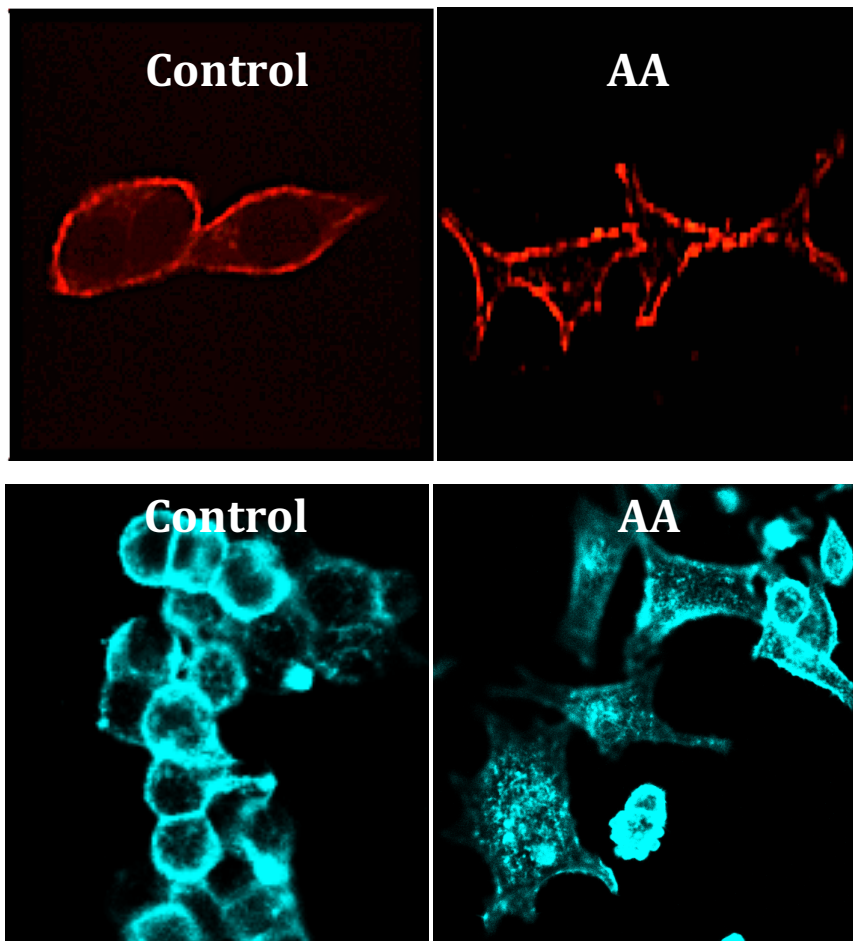

Supplementary Figure 3

Supplement: Supplementary file 3 — Additional file 3: Figure S3. Morphological changes induced in BON cells by overnight treatment with 6 µM AA. Cell shapes were visualized by treatment with phallotoxin fluorescently-labeled with Alexa Fluor 546 (red) or 633 (cyan). Confocal images of cells in culture are shown following treatment with vehicle contro (DMSO) or AA as indicated. [file 12935_2018_529_MOESM3_ESM.pdf]
